# Insight into systematic formation of hexafluorosilicate during crystallization *via* self-assembly in a glass vessel†

**DOI:** 10.1039/d2ra04270c

**Published:** 2022-09-05

**Authors:** Dongwon Kim, Jihun Han, Ok-Sang Jung, Young-A. Lee

**Affiliations:** Department of Chemistry, Pusan National University Busan 46241 Republic of Korea oksjung@pusan.ac.kr Fax: (+82) 51-5163522 (+82) 51-5103240; Department of Chemistry, Jeonbuk National University Jeonju 54896 Korea ylee@jbnu.ac.kr

## Abstract

Formation of the unexpected hexafluorosilicate (SiF_6_^2−^) anion during crystallization *via* self-assembly in glassware is scrutinized. Self-assembly of M(BF_4_)_2_ (M^2+^ = Cu^2+^ and Zn^2+^) with tridentate N-donors (L) in a mixture solvent including methanol in a glass vessel gives rise to an SiF_6_^2−^-encapsulated Cu_3_L_4_ double-decker cage and a Zn_2_L_4_ cage, respectively. Induced reaction of CuX_2_ (X^−^ = PF_6_^−^ and SbF_6_^−^) instead of Cu(BF_4_)_2_, with the tridentate ligands, produces the same species. The formation time of SiF_6_^2−^ is in the order of anions BF_4_^−^ < PF_6_^−^ < SbF_6_^−^ under the given reaction conditions. The SiF_6_^2−^ anion, acting as a cage template or cage-to-cage bridge, seems to be formed from the reaction of polyatomic anions containing fluoride with the SiO_2_ of the surface of the glass vessel.

## Abstract 2

Formation of the unexpected hexafluorosilicate (SiF_6_^2−^) encapsulated cages constructed. Interestingly, this shows that the surface of glassware should be given serious consideration for long-duration reactions with active F-containing species.

## Graphical abstract

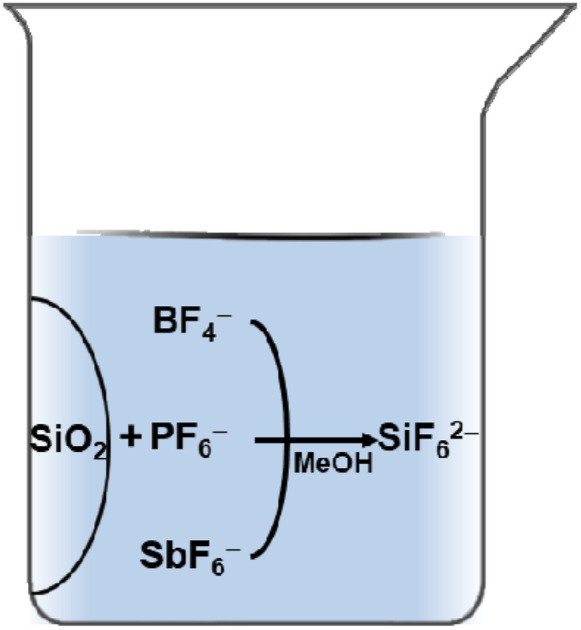

## Introduction

Anion-related chemistry is a rapidly burgeoning field owing to the timeliness and importance including environmental pollution, industrial chemicals, biological processes, ionic liquids, lithium batteries, templates, ion-pairing, and health.^1–12^ Some anions play a significant role in the construction and behaviors of desirable molecular architectures based on negative charge, size, shape, solvents, and pH.^13–18^ In particular, F-containing polyatomic anions such as BF_4_^−^, PF_6_^−^, and SbF_6_^−^ have been extensively employed for various purposes such as important chemical phenomena, cage-templates, coordination, and the Hofmeister series;^19–26^ however, they are known to be not thermodynamically stable depending on conditions^27,28^ relative to the F-free polyatomic anions NO_3_^−^, ClO_4_^−^, CH_3_CO_2_^−^, and SO_4_^2−^. For instance, the PF_6_^−^ anion could be converted to PF_*n*_(OH)_6−*n*_^−^ during the complexation of Pd(ii)-L.^29^ Furthermore, BF_4_^−^ is known to be changed to the SiF_6_^2−^ anion *via* subsequent reaction of dissociated F^−^ with the Si–O moieties of a glass vessel or stoneware.^30–35^ Hexafluorosilicate (SiF_6_^2−^) is a stable octahedral anion with six Si–F bond distances of 1.71 Å,^36^ and its solubility is significantly dependent on cations.^37^ Furthermore, the dianion species have seen the following diverse uses: leather and wood preservation agents, fluoridation agents for drinking water, two-solution fluoride mouth rinse, commercial laundry, enamels/enamel frits for china and porcelain, opalescent glass, metallurgy of aluminum and beryllium, glue, ore flotation, insecticides and rodenticides, fluorinating agent in organic synthesis, and water fluoridation.^38–47^

In this context, systematic research on the unusual formation process and recognition of SiF_6_^2−^ is very much in demand. Thus, herein, formation of hexafluorosilicate *via* conversion of PF_6_^−^ and SbF_6_^−^ beside BF_4_^−^ during self-assembly or recrystallization in a glass vessel is scrutinized. This paper reports the unique SiF_6_^2−^-encapsulated cages constructed *via* self-assembly of M^2+^ (M = Cu^2+^ and Zn^2+^) with tridentate N-donor ligands in a glass vessel. To our knowledge, this article presents clear systematic research on the formation of hexafluorosilicate by reactions of glassware with F-containing polyatomic anions.

## Experimental

### Materials and measurements

All of the chemicals, including copper(ii) tetrafluoroborate (Cu(BF_4_)_2_), copper(ii) nitrate (Cu(NO_3_)_2_), copper(ii) chloride (CuCl_2_), silver(i) hexafluorophosphate (AgPF_6_), silver(i) hexafluoroantimonate (AgSbF_6_), and zinc(ii) tetrafluoroborate (Zn(BF_4_)_2_), were purchased from Aldrich and used without further purification. (1*S*,1′*S*,1′′*S*,2*R*,2′*R*,2′′*R*)-(Benzenetricarbonyltris(azanediyl))tris(2,3-dihydro-1H-indene-2,1-diyl)triisonicotinate (L^1^) and 1,3,5-tris(isonicotinoyloxy-methyl)benzene (L^2^) were synthesized according to the literature,^48,49^ respectively. Glass vessels (Hubena Co, Seongnam, Korea) were employed in all of the self-assembly reactions. The ^1^H and ^19^F NMR spectra were recorded on a Varian Mercury Plus operating at 400 MHz. The infrared (IR) spectra were obtained on a Nicolet 380 FT-IR spectrophotometer with samples prepared as KBr pellets. Elemental microanalyses (C, H, N) were performed on solid samples at the Pusan Center, KBSI, using a Vario-EL III. Thermal analyses were carried out under a dinitrogen atmosphere at a scan rate of 10°C min^−1^ using a Labsys TGA-DSC 1600.

### [(SiF_6_)_2_@Cu_3_L^1^_4_](SiF_6_)·16CH_3_OH

#### Method 1

A methanol solution (2.0 mL) of copper(ii) tetrafluoroborate (4.7 mg, 0.02 mmol) was carefully layered onto a dichloromethane solution (2.0 mL) of L^1^ (18.4 mg, 0.02 mmol). After 2 weeks, blue crystals suitable for single crystal X-ray structure determination were obtained in an 83% yield.

#### Method 2

A methanol solution (1.0 mL) of silver(i) hexafluorophosphate (10.1 mg, 0.04 mmol) was added to copper(ii) chloride (2.7 mg, 0.02 mmol) dispersed in methanol at room temperature. The reaction mixture was stirred for 30 min, after which, the precipitated silver chloride was filtered off. The methanol solution of Cu(PF_6_)_2_ was carefully layered onto a dichloromethane solution (2.0 mL) of L^1^ (18.4 mg, 0.02 mmol). After 4 weeks, blue crystals suitable for single crystal X-ray structure determination were obtained in a 78% yield.

#### Method 3

A methanol solution (1.0 mL) of silver(i) hexafluoroantimonate (13.7 mg, 0.04 mmol) was added to a suspension of copper(ii) chloride (2.7 mg, 0.02 mmol) dispersed in methanol at room temperature. The reaction mixture was stirred for 30 min, after which, the precipitated silver chloride was filtered off. The methanol solution of Cu(SbF_6_)_2_ was carefully layered onto a dichloromethane solution (2.0 mL) of L^1^ (18.4 mg, 0.02 mmol). After 6 weeks, blue crystals suitable for single crystal X-ray structure determination were obtained in a 66% yield.

#### Method 4

A methanol solution (2.0 mL) of copper(ii) nitrate (2.8 mg, 0.015 mmol), a dichloromethane solution (4.0 mL) of L^1^ (18.4 mg, 0.02 mmol), and an aqueous solution (0.1 mL) of (NH_4_)_2_SiF_6_ (3.6 mg, 0.02 mmol) were left at room temperature, and after 1 day, blue crystals suitable for single crystal X-ray structure determination were obtained in a 62% yield. m. p. 280 °C (dec). Anal. Calcd for: C, 58.55; H, 4.65; N, 7.19%. Found: C, 58.40; H, 4.52; N, 7.23%. IR (KBr pellet, cm^−1^): 3294(br), 1733(s), 1661(s), 1524(s), 1425(m), 1329(m), 1286(s), 1179(w), 1127(s), 1063(m), 1032(m), 859(w), 756(s), 705(s), 472(w).

### [(SiF_6_)@Zn_2_L^2^_4_](SiF_6_)·2C_4_H_8_O·4CH_2_Cl_2_

A mixture of tetrahydrofuran with a methanol solution (3.0 mL/1.0 mL) of zinc(ii) tetrafluoroborate (9.6 mg, 0.04 mmol) and a dichloromethane solution (3.0 mL) of L^2^ (14.5 mg, 0.04 mmol) were left at room temperature, and after 2 weeks, blue crystals suitable for single crystal X-ray structure determination were obtained in a 67% yield. m. p. 260 °C (dec). Anal. Calcd for: C, 50.88; H, 3.84; N, 5.93%. Found: C, 50.79; H, 3.81; N, 5.97%. IR (KBr pellet, cm^−1^): 3425(br), 1729(s), 1616(w), 1564(w) 1409(m), 1373(w) 1326(m), 1280(s), 1224(w), 1122(s), 1062(m), 1024(w), 862(m), 759(s), 705(s), 468(w).

### Single crystal X-ray structure determination

The X-ray diffraction data for [(SiF_6_)_2_@Cu_3_L^1^_4_](SiF_6_)·16CH_3_OH were measured at 100 K with synchrotron radiation (*λ* = 0.76000 Å) on a Rayonix MX225HS detector at 2D SMC with a silicon (111) double-crystal monochromator (DCM) at the Pohang Accelerator Laboratory (PAL), Korea. The PAL BL2D-SMDC program^50^ was used for data collection (detector distance: 66 mm, omega scan: Δ*ω* = 3°, exposure time: 1 s per frame), and HKL3000sm (ver. 703r)^51^ was used for cell refinement, reduction, and absorption correction. X-ray data on [(SiF_6_)@Zn_2_L^2^_4_](SiF_6_)·2C_4_H_8_O·4CH_2_Cl_2_ were collected on a Bruker SMART automatic diffractometer with graphite-monochromated Mo K*α* radiation (*λ* = 0.71073 Å). Thirty-six (36) frames of 2D diffraction images were collected and processed to obtain the cell parameters and orientation matrix. The data were corrected for Lorentz and polarization effects. The absorption effects were corrected using the multi-scan method (SADABS).^52^ The structures were resolved using the direct method (SHELXS) and refined by full-matrix least squares techniques (SHELXL 2018/3).^53^ The non-hydrogen atoms were refined anisotropically, and the hydrogen atoms were placed in calculated positions and refined only for the isotropic thermal factors. The crystal parameters and procedural information corresponding to the data collection and structure refinement are listed in Table 1.

**Table 1:** Selected bond lengths (Å) and angles (°) for [(SiF_6_)_2_@Cu_3_L^1^_4_](SiF_6_)·16CH_3_OH, and [(SiF_6_)@Zn_2_L^2^_4_](SiF_6_)·2C_4_H_8_O·4CH_2_Cl_2_

[(SiF_6_)_2_@Cu_3_L^1^_4_](SiF_6_)·16CH_3_OH		[(SiF_6_)@Zn_2_L^2^_4_](SiF_6_)·2C_4_H_8_O·4CH_2_Cl_2_	
Cu(1)–N(6)	2.007(4)	Zn(1)–N(1)	2.115(4)
Cu(1)–N(3)	2.019(3)	Zn(1)–F(4)	2.122(5)
Cu(1)–F(5)	2.317(2)	Zn(1)–F(3)	2.361(5)
N(6)^a^–Cu(1)–N(6)	87.5(2)	N(1)–Zn(1)–N(1)^a^	89.952(6)
N(6)^a^–Cu(1)–N(3)^a^	176.4(3)	N(1)^a^–Zn(1)–N(1)^b^	176.7(2)
N(6)–Cu(1)–N(3)^a^	91.2(2)	N(1)^a^–Zn(1)–N(1)^c^	89.953(6)
N(6)–Cu(1)–N(3)	176.4(2)	N(1)–Zn(1)–F(4)	91.6(1)
N(3)^a^–Cu(1)–N(3)	90.3(3)	N(1)–Zn(1)–F(3)	88.4(1)
		F(4)–Zn(1)–F(3)	180.0

a *x* − 1, *y* + 1, *z*, *x* + 1, *y* − 1, *z*.

b *x* + 1, *y*, *z* − 1.

c −*x* + 1/2, −*y* + 1/2, *z*

## Results and discussion

### Synthesis aspects

**Scheme 1:**
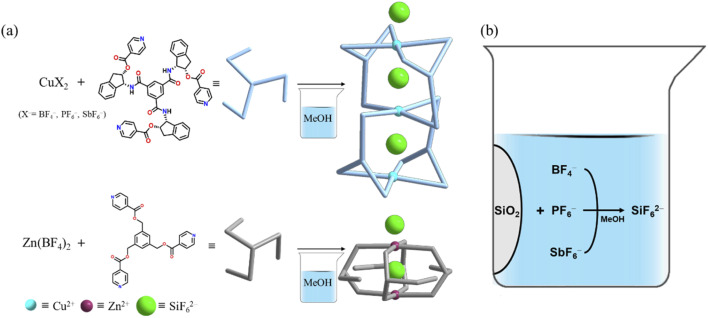
Construction of cages (a) *via* SiF_6_^2−^ formation (b) during self-assembly in a glass vessel.

Self-assembly of Cu(BF_4_)_2_ with L^1^ in a mixture of methanol and dichloromethane produced crystals consisting of [(SiF_6_)_2_@Cu_3_L^1^_4_](SiF_6_)·16CH_3_OH suitable for single crystal X-ray structure determination after 2 weeks, as shown in Scheme 1. Moreover, the reactions with Cu(PF_6_)_2_ and Cu(SbF_6_)_2_ instead of Cu(BF_4_)_2_ gave rise to the same product after significant durations, specifically 4 and 6 weeks, respectively. The reaction time may be owed to the stability of the BF_4_^−^ < PF_6_^−^ < SbF_6_^−^ anions under the given reaction conditions. As expected, self-assembly of Cu(BF_4_)_2_ with L^1^ in the presence of (NH_4_)_2_SiF_6_ produced blue crystals within 1 day. In order to investigate the metal effects, self-assembly of Zn(BF_4_)_2_ instead of Cu(BF_4_)_2_ with L^2^ was performed, which generated crystals of [(SiF_6_)@Zn_2_L^2^_4_](SiF_6_)·2C_4_H_8_O·4CH_2_Cl_2_ after 2 weeks, indicating that the central metal cation is not a significant factor in anion transformation in a glass vessel. For all of the reactions, the SiF_6_^2−^ anion acted either as a template by which to form the cage products or as a cage-to-cage bridge.

Both crystalline products are stable under anaerobic condition at room temperature. The crystals are insoluble in common organic solvents such as acetone, chloroform, toluene, benzene and tetrahydrofuran, but are partially dissociated in dimethyl sulfoxide and *N*,*N*-dimethylformamide. Their compositions and structures were confirmed by elemental analyses, IR-spectral analysis (Fig. S1 and S2†), thermal analysis (Fig. S3†), ^1^H NMR (Fig. S4, as dissoociated in Me_2_SO-*d*_6_†), and single-crystal X-ray crystallography. The characteristic IR bands of SiF_6_^2−^ were easily assigned. The thermogravimetric analysis (TGA) and differential scanning calorimetry (DSC) showed [(SiF_6_)_2_@Cu_3_L^1^_4_](SiF_6_)·16CH_3_OH and [(SiF_6_)@Zn_2_L^2^_4_](SiF_6_)·2C_4_H_8_O·4CH_2_Cl_2_ to be stable up to 277 and 257 °C, respectively. Evaporation of the solvate molecules of the two kinds of crystals occurred in the 10–60 and 20–135 °C temperature ranges, respectively (Fig. S3†).

### Crystal structures

**Fig. 1:**
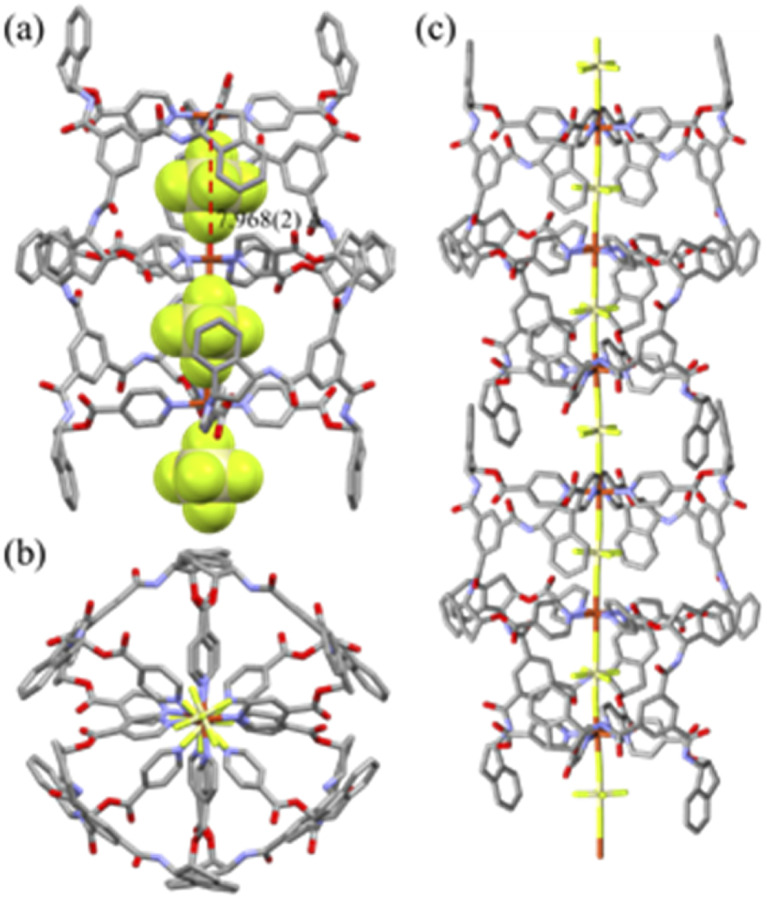
Side view (a), top view (b), and packing structure (c) of double-decker, [(SiF_6_)_2_@Cu_3_L^1^_4_](SiF_6_)·16CH_3_OH. The nestled and outside bridged SiF_6_^2−^ anions were clearly depicted in light green color.

**Fig. 2:**
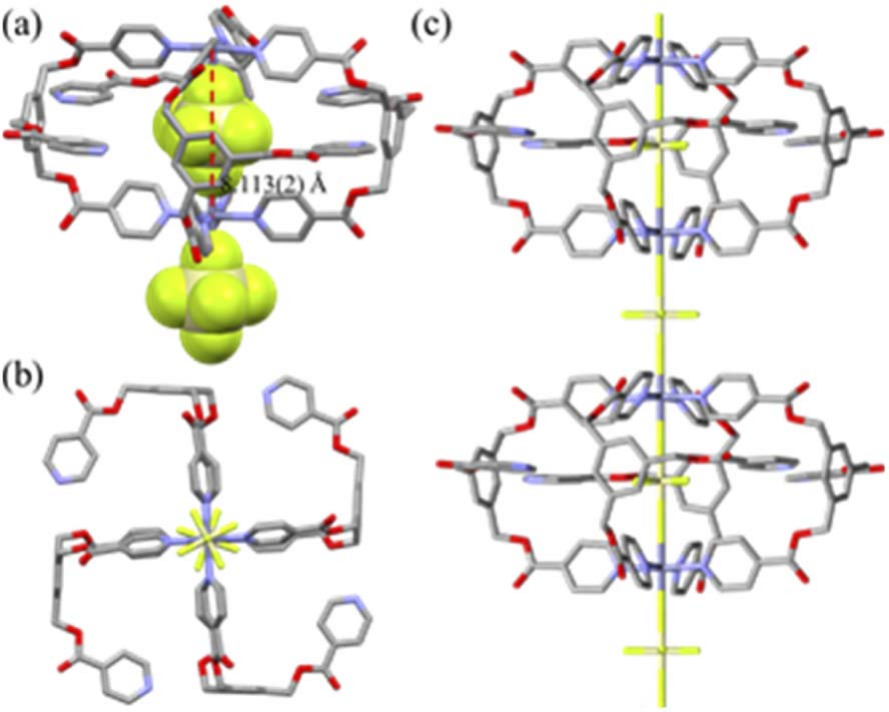
Partial crystal structures of [Ag(*s*,*r*-L)](PF_6_)·3C_4_H_8_O_2_·0.5H_2_O (a) and [Ag(*r*,*s*-L)](PF_6_)·3C_4_H_8_O_2_·0.5H_2_O (b). The solvate molecules were omitted for clarity.

The crystal structure of [(SiF_6_)_2_@Cu_3_L^1^_4_](SiF_6_)·16CH_3_OH is depicted in Fig. 1, and its bond lengths and angles are listed in Table 1. The geometry of each copper(ii) ion is an octahedral N_4_F_2_ coordination arrangement with four N donors from four L^1^s and F donors from two SiF_6_^2−^ anions (Cu–N = 2.007(4) − 2.019(3) Å; Cu–F(encapsulated) = 2.317(2), 2.300(3) Å; Cu–F(bridged) = 2.357(3) Å) in axial positions, resulting in the formation of the Cu_3_L_4_ double-decker occupied by two SiF_6_^2−^ anions. Concomitantly, for the nestled SiF_6_^2−^, the equatorial Si–F bond lengths (1.628(7) − 1.684(7) Å) are comparable to the axial Si–F lengths (1.666(3) and 1.693(3) Å). Thus, the ligand is coordinated to two Cu(ii) ions in a bidentate and a monodentate fashion, respectively. The intra-cage Cu⋯Cu distance is 7.968(2) Å. Its packing structure is in the linear double-decker⋯SiF_6_^2−^⋯double-decker⋯SiF_6_^2−^ mode (Fig. S5†). The crystal structure of [(SiF_6_)@Zn_2_L^2^_4_](SiF_6_)·2C_4_H_8_O·4CH_2_Cl_2_ is a typical Zn_2_L_4_ cage, as depicted in Fig. 2, and the coordination geometry of the Zn(ii) ion is an octahedral arrangement with two SiF_6_^2−^ groups in the *trans* position (Zn–F = 2.095(5) − 2.273(5) Å; F–Zn–F′ = 180.0(0)°) and four pyridine N donors of four L^2^s on the basal plane. Each L connects two zinc(ii) ions in a bidentate mode with a free pyridyl donor. Concomitantly, for the nestled SiF_6_^2−^, the equatorial Si–F bond lengths (1.637(4) Å) are slightly shorter than the axial Si–F lengths (1.733(5) – 1.746(6) Å). The intra-cage Zn⋯Zn distance is 8.113(2) Å. Its packing structure is in the linear cage⋯SiF_6_^2−^⋯ cage⋯SiF_6_^2−^ mode (Fig. S5†). The solvate molecules of [(SiF_6_)_2_@Cu_3_L^1^_4_](SiF_6_)·16CH_3_OH were squeezed. The volumes of solvate molecules in [(SiF_6_)_2_@Cu_3_L^1^_4_](SiF_6_)·16CH_3_OH and [(SiF_6_)@Zn_2_L^2^_4_](SiF_6_)·2C_4_H_8_O·4CH_2_Cl_2_ were 56.2% (10 282 Å^3^/18 304 Å^3^) and 37.8% (2093.4 Å^3^/5539.5 Å^3^), respectively, on the basis of a PLATON/SOLV calculation.^54^

## Discussion

Formation of SiF_6_^2−^ during self-assembly of more than 2 weeks' duration requires some explanation, because the anion was not added to the self-assembly solution in a glass vessel. It should be noted that the SiF_6_^2−^ anion replaces the starting BF_4_^−^ anion in the self-assembly reaction and that it is formed after extracting Si^4+^ from the glass vessel in methanol solvent—a rare but well-known phenomenon.^55^ The generation of SiF_6_^2−^ by slow release of Si^4+^ from the glass vessel in the methanol solution was clearly confirmed, and the resultant anions were separated as counteranions. The SiF_6_^2−^ anions appear to play significant roles as both encapsulator and bridge in the construction of the cage skeleton. The stable anion, by contrast, forms as a consequence of the hydrolysis of the BF_4_^−^ anion and subsequent reaction of F^−^ with the Si–O moieties of the glass vessel^56,57^ in which a methanol solution of M(BF_4_)_2_ is kept for self-assembly or crystallization. The formation of SiF_6_^2−^ seems to be promoted by the presence of a small quantity of H_2_O in addition to a mixture solvent including methanol. Formation of SiF_6_^2−^ from BF_4_^−^ and PF_6_^−^ in a glass vessel in water has been observed by other groups,^30–32,56^ but that of SiF_6_^2−^ from SbF_6_^−^ heretofore has not been recorded. The present self-assembly of MX_2_ (M^2+^ = Cu^2+^, Zn^2+^; X^−^ = BF_4_^−^, PF_6_^−^, SbF_6_^−^) with each tridentate ligand in each glass vessel systematically transformed BF_4_^−^, PF_6_^−^ or SbF_6_^−^ into the SiF_6_^2−^ anion according to the following eqn (1):

1 SiO_2_ + 6HF → SiF_6_^2−^ + 2H_3_O^+^

Of course, formation of SiF_6_^2−^ from CF_3_SO_3_^−^ (C–F compound) could not be carried out in a similar reaction performed in a polypropylene vessel instead of a glass one; that is, the reaction did not yield crystals containing the SiF_6_^2−^ anion.

The presence of SiF_6_^2−^ in Me_2_SO-*d*^6^ was confirmed by ^19^F NMR spectra (Fig. S6†). The SiF_6_^2−^ occurred as a singlet at *δ* = −134 ppm. Spectra taken of the sample preparation one month later indicated that almost all of the original BF_4_^−^ anions had been converted to SiF_6_^2−^ anions. As regards the formation of SiF_6_^2−^ from PF_6_^−^ and SbF_6_^−^ anions, PF_6_^−^ (−70.8 ppm) and SbF_6_^−^ chemical shifts (−119.9 ppm) disappeared after 4 and 6 weeks, respectively, and appeared at the position of the SiF_6_^2−^ anion. As for construction of the coordination architecture, insight into the stability and geometry of a specific anion as a template is useful to the tuning of the shape and topology of coordination species. In the present study, the encapsulated and bridged SiF_6_^2−^ anions helped to stabilize the crystal structure of the cages. Certainly, transformation of polyatomic anions and their role is a topic worthy of further investigation.

## Conclusions

In summary, transformation of polyatomic anions BF_4_^−^, PF_6_^−^, and SbF_6_^−^ into SiF_6_^2−^ during self-assembly or recrystallization in a glass vessel was systematically confirmed. This shows that the surface of regular laboratory glassware should be given serious consideration for long-duration reactions or self-assembly with F^−^ species. Further studies on other means of anion-bridging or -encapsulation of cage units will contribute to the development of task-specific polyatomic anions for recognition as well as environmental molecular materials such as adsorbents and sensing materials.

## Conflicts of interest

There are no conflicts to declare.

## Supplementary Material

RA-012-D2RA04270C-s001

RA-012-D2RA04270C-s002

## References

[cit1] Anthony J. L., Anderson J. L., Maginn E. J., Brennecke J. F. (2005). J. Phys. Chem. B.

[cit2] Wang Z., Li X., Guo W., Fu Y. (2021). Adv. Funct. Mater..

[cit3] Carpenter J. P., McTernan C. T., Ronson T. K., Nitschke J. R. (2019). J. Am. Chem. Soc..

[cit4] Gale P. A., Perez-Tomas R., Quesada R. (2013). Acc. Chem. Res..

[cit5] Shin D. H., Kim M., Kim Y., Jun I., Jung J., Nam J. H., Cheng M. H., Lee M. G. (2020). Pflugers Arch. - Eur. J. Physiol..

[cit6] Zhao Y., Li Y., Yuan S., Zhu J., Houtmeyers S., Li J., Dewil R., Gao C., Van der Bruggen B. (2019). J. Mater. Chem. A.

[cit7] Zhao Y., Cotelle Y., Liu L., López-Andarias J., Bornhof A.-B., Akamatsu M., Sakai N., Matile S. (2018). Acc. Chem. Res..

[cit8] Bando Y., Haketa Y., Sakurai T., Matsuda W., Seki S., Takaya H., Maeda H. (2016). Chem. Eur. J..

[cit9] Patnaik S., Sahoo D. P., Parida K. (2021). Carbon.

[cit10] Kim Y., Kang P., Jeon Y., Cho H. M., Choi M.-G. (2021). Bull. Korean Chem. Soc..

[cit11] Sohn D. H., Ohn T., Han E., Atar A. B., Cho S. J., Kang J. (2021). Bull. Korean Chem. Soc..

[cit12] Lee J., Hong Y., Yeon S., Moon D., You T.-S. (2021). Bull. Korean Chem. Soc..

[cit13] Xu Z., Chen X., Chen R., Li X., Zhu H. (2020). Npj Comput. Mater..

[cit14] Liu D., Wang J., Wang Y., Zhu Y. (2018). Catal. Sci. Technol..

[cit15] Liu J.-J., Xia S.-B., Duan Y.-L., Liu T., Cheng F.-X., Sun C.-K. (2018). Polymers.

[cit16] Li G.-B., Zhang Z., Liao L.-S., Pan R.-K., Liu S.-G. (2021). Spectrochim. Acta, Part A.

[cit17] Caballero A., Zapata F., Beer P. D. (2013). Coord. Chem. Rev..

[cit18] Wong J. E., Zastrow H., Jaeger W., von Klitzing R. (2009). Langmuir.

[cit19] He X., Zhang K., Liu Y., Wu F., Yu P., Mao L. (2018). Angew. chem..

[cit20] Sarada G., Kim A., Kim D., Jung O.-S. (2020). Dalton Trans..

[cit21] Lee J., Lim S., Kim D., Jung O.-S., Lee Y.-A. (2020). Dalton Trans..

[cit22] Noro S.-i., Nakamura T. (2017). NPG Asia Mater..

[cit23] Takeuchi M., Matubayasi N., Kameda Y., Minofar B., Ishiguro S.-i., Umebayashi Y. (2012). J. Phys. Chem. B.

[cit24] Pramanik A., Powell D. R., Wong B. M., Hossain M. A. (2012). Inorg. Chem..

[cit25] Kim D., Gwak G., Han J., Kim D., Jung O.-S. (2022). Dalton Trans..

[cit26] Jaganathan J., Sivapragasam M., Wilfred C. (2016). J. Chem. Eng. Process Technol..

[cit27] Swatloski R. P., Holbrey J. D., Rogers R. D. (2003). Green Chem..

[cit28] Reed C. A. (2011). Chem. N. Z..

[cit29] Noh T. H., Lee H., Lee Y.-A., Jung O.-S. (2013). Inorg. Chim. Acta.

[cit30] Sun M.-Y., Wang X.-Z., Chen Z.-Y., Zhou X.-P., Li D. (2019). Inorg. Chem..

[cit31] Mizuhata M., Saito Y., Takee M., Deki S. (2009). J. Ceram. Soc. Jpn..

[cit32] Reger D. L., Pascui A. E., Smith M. D. (2012). Eur. J. Inorg. Chem..

[cit33] Galstyan A., Shen W. Z., Freisinger E., Alkam H., Hiller W., Sanz Miguel P. J., Schürmann M., Lippert B. (2011). Chem. Eur. J..

[cit34] Chen W., Chu J., Mutikainen I., Reedijk J., Turpeinen U., Song Y.-F. (2011). CrystEngComm.

[cit35] Nielsen M. M., Pedersen C. M. (2022). Chem. Sci..

[cit36] Wiberg E. , Holleman A. F. and Wiberg N., Inorganic chemistry, Academic press, 2001

[cit37] Frayret J., Castetbon A., Trouve G., Potin-Gautier M. (2006). Chem. Phys. Lett..

[cit38] Bianchetti G. O., Devlin C. L., Seddon K. R. (2015). RSC Adv..

[cit39] Urbansky E., Schock M. (2000). Int. J. Environ. Sci..

[cit40] Amouri H., Desmarets C., Moussa J. (2012). Chem. Rev..

[cit41] Shindo M., Iwata T. (2022). Synlett.

[cit42] Thompson C. M. , Dianion chemistry in organic synthesis, CRC press, 1994

[cit43] Hudnall T. W., Chiu C.-W., Gabbai F. P. (2009). Acc. Chem. Res..

[cit44] Derkson G., Poon P., Richardson A. (1982). J. Dent. Res..

[cit45] Chow L. C., Takagi S., Carey C. M., Sieck B. (2000). J. Dent. Res..

[cit46] Sharma R. P., Sharma R., Bala R., Rychlewska U., Warzajtis B., Ferretti V. (2005). J. Mol. Struct..

[cit47] Lusher J., Sebba F. (1966). J. Appl. Chem..

[cit48] Kim D., Seo K.-D., Shim Y.-B., Lee K., Lee S. H., Lee Y.-A., Jung O.-S. (2022). Dalton Trans..

[cit49] Choi D., Lee H., Lee J. J., Jung O.-S. (2017). Cryst. Growth Des..

[cit50] Shin J. W., Eom K., Moon D. (2016). J. Synchrotron Radiat..

[cit51] Otwinowski Z. and Minor W., Methods in Enzymology, ed. C. W. Carter Jr. and R. M. Sweet, Academic Press, New York, 1997, 276, p. 307 10.1016/S0076-6879(97)76066-X 27754618

[cit52] Sheldrick G. M. S. A. D. A. B. S. , A program for Empirical Absorption Correction of Area Detector Data, University of Göttingen, Germany, 1996

[cit53] Sheldrick G. M. (2015). Acta Crystallogr., Sect. C: Struct. Chem..

[cit54] Spek A. (2009). Acta Crystallogr., Sect. D: Biol. Crystallogr..

[cit55] Mysen B. O., Virgo D. (1986). Chem. Geol..

[cit56] Wu J. Y., Zhong M. S., Chiang M. H. (2017). Chem. Eur. J..

[cit57] Jia J., Blake A. J., Champness N. R., Hubberstey P., Wilson C., Schroder M. (2008). Inorg. Chem..

