# Supplementary material for: Insight into systematic formation of hexafluorosilicate during crystallization via self-assembly in a glass vessel
Source: RSC Adv. 2022 Sep 5;12(39):25118–22. doi: 10.1039/d2ra04270c (PMC9443674; doi:10.1039/d2ra04270c)
Supplement: RA-012-D2RA04270C-s001 [file RA-012-D2RA04270C-s001.pdf]

Electronic Supplementary Material (ESI) for RSC Advances.
This journal is © The Royal Society of Chemistry 2022

<Electronic Supplementary Information>

Insight into systematic formation of hexafluorosilicate during crystallization via self-assembly in glass vessel

Dongwon Kim,[a] Jihun Han,[a] Ok-Sang Jung,[*a] and Young-A Lee[*b]

[a]Department of Chemistry, Pusan National University, Busan 46241, Republic of Korea

E-mail: oksjung@pusan.ac.kr

[b]*Department of Chemistry, Jeonbuk National University, Jeonju 54896, Republic of Korea*

E-mail: ylee@jbnu.ac.kr

Table S1 Crystallographic Data for $[(SiF_6)_2@Cu_3L^1{}_4](SiF_6)\cdot16CH_3OH$ and $[(SiF_6)@Zn_2L^2{}_4](SiF_6)\cdot2C_4H_8O\cdot4CH_2Cl_2$.

	$[(SiF_6)_2@Cu_3L^1{}_4](SiF_6)\cdot16CH_3OH$	$[(SiF_6)@Zn_2L^2{}_4](SiF_6)\cdot2C_4H_8O\cdot4CH_2Cl_2$
Formula	$C_{216}H_{168}Cu_3F_{18}N_{24}O_{36}Si_3$	$C_{60}H_{54}Cl_4F_6N_6O_{13}SiZn$
M_w	4292.62	1416.35
Cryst. sys.	Monoclinic	Tetragonal
Space group	*C*2	*P*4/n
a (Å)	38.961(8)	20.713(2)
b (Å)	21.921(4)	20.713(2)
c (Å)	23.987(5)	15.855(1)
α (°)	90	90
β (°)	116.69(3)	90
γ (°)	90	90
V (Å^3)	18304(8)	6803(1)
Z	2	4
ρ (g cm^{-3})	0.779	1.383
μ (mm^{-1})	0.281	0.615
F(000)	4422	2904
Independent reflections (R_{int})	32740 (0.0749)	6691 (0.0807)
GooF	0.786	1.037
Absolute structure parameter	0.020(5)	-
Final R indices [I>2σ(I)]	R_1 = 0.0412, wR_2 = 0.0906	R_1 = 0.0633, wR_2 = 0.1956
R indices (all data)	R_1 = 0.1158, wR_2 = 0.1100	R_1 = 0.0963, wR_2 = 0.2327
Largest diff. peak and hole (e/Å^3)	0.224 and -0.244	1.500 and -0.620

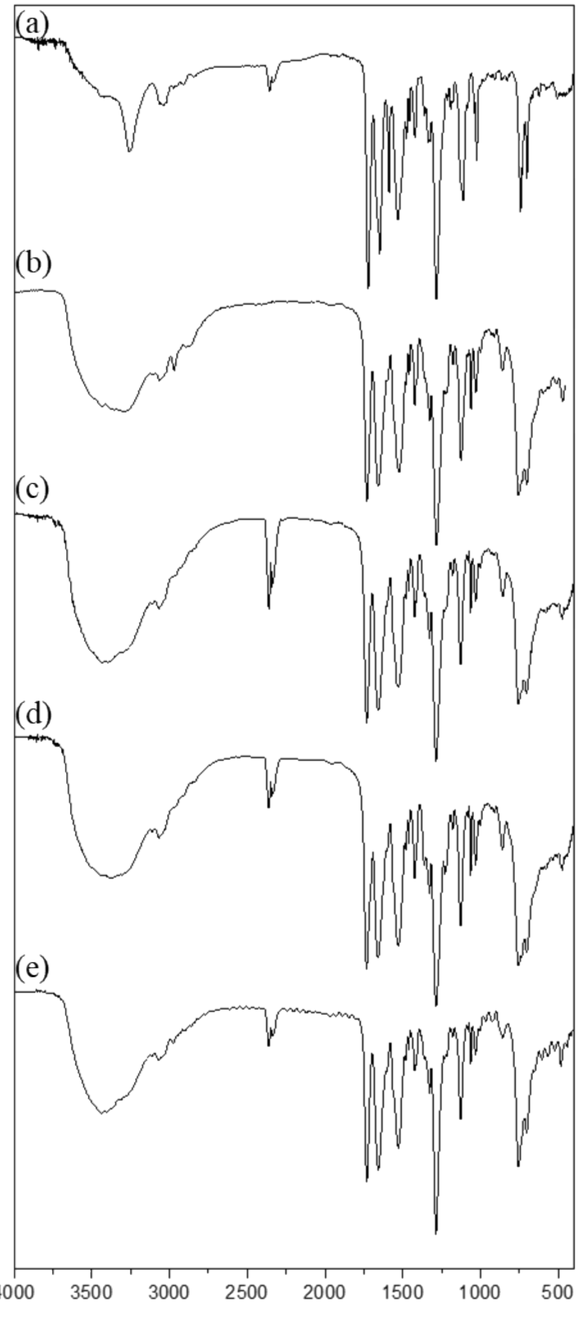

Fig. S1 IR spectra for L^1 (a), $[(SiF_6)_2@Cu_3L^1{}_4](SiF_6)\cdot16CH_3OH$ using *Method 1* (b), $[(SiF_6)_2@Cu_3L^1{}_4](SiF_6)\cdot16CH_3OH$ using *Method 2* (c), $[(SiF_6)_2@Cu_3L^1{}_4](SiF_6)\cdot16CH_3OH$ using *Method 3* (d), and $[(SiF_6)_2@Cu_3L^1{}_4](SiF_6)\cdot16CH_3OH$ using *Method 4* (e).

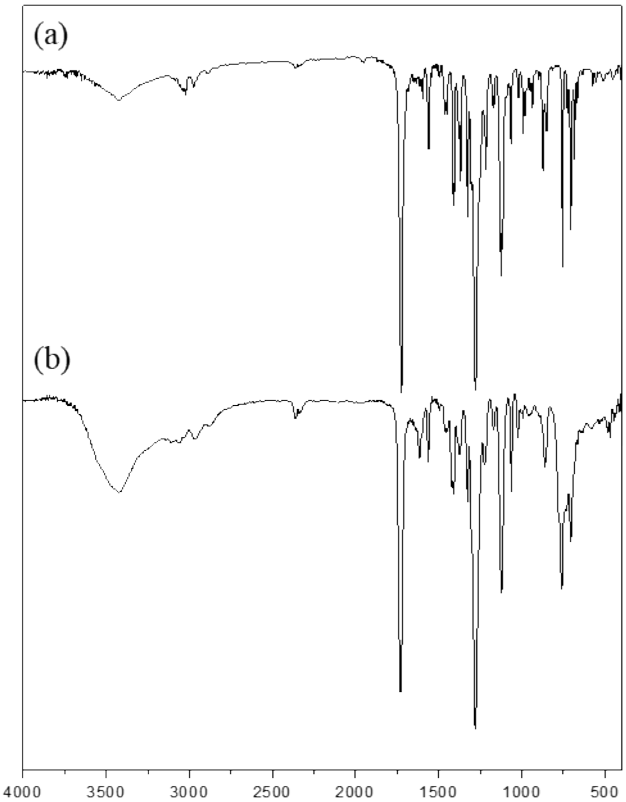

Fig. S2 IR spectra for L^2 (a), and $[(SiF_6)@Zn_2L^2{}_4](SiF_6)\cdot 2C_4H_8O\cdot 4CH_2Cl_2$ (b).

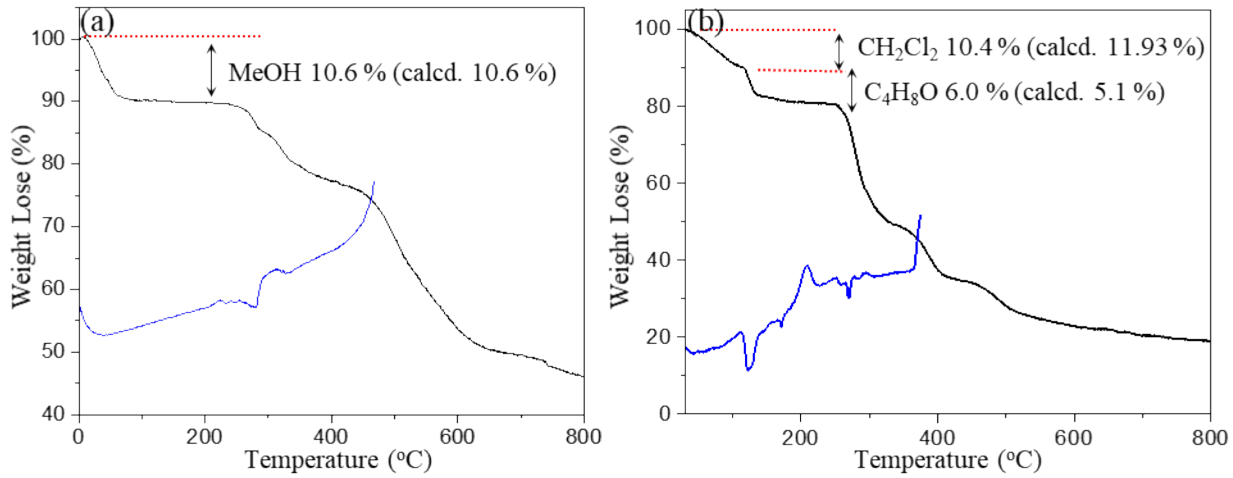

Fig. S3 TG and DSC curves for $[(SiF_6)_2@Cu_3L^1{}_4](SiF_6)\cdot 16CH_3OH$ (a), and $[(SiF_6)@Zn_2L^2{}_4](SiF_6)\cdot 2C_4H_8O\cdot 4CH_2Cl_2$ (b).

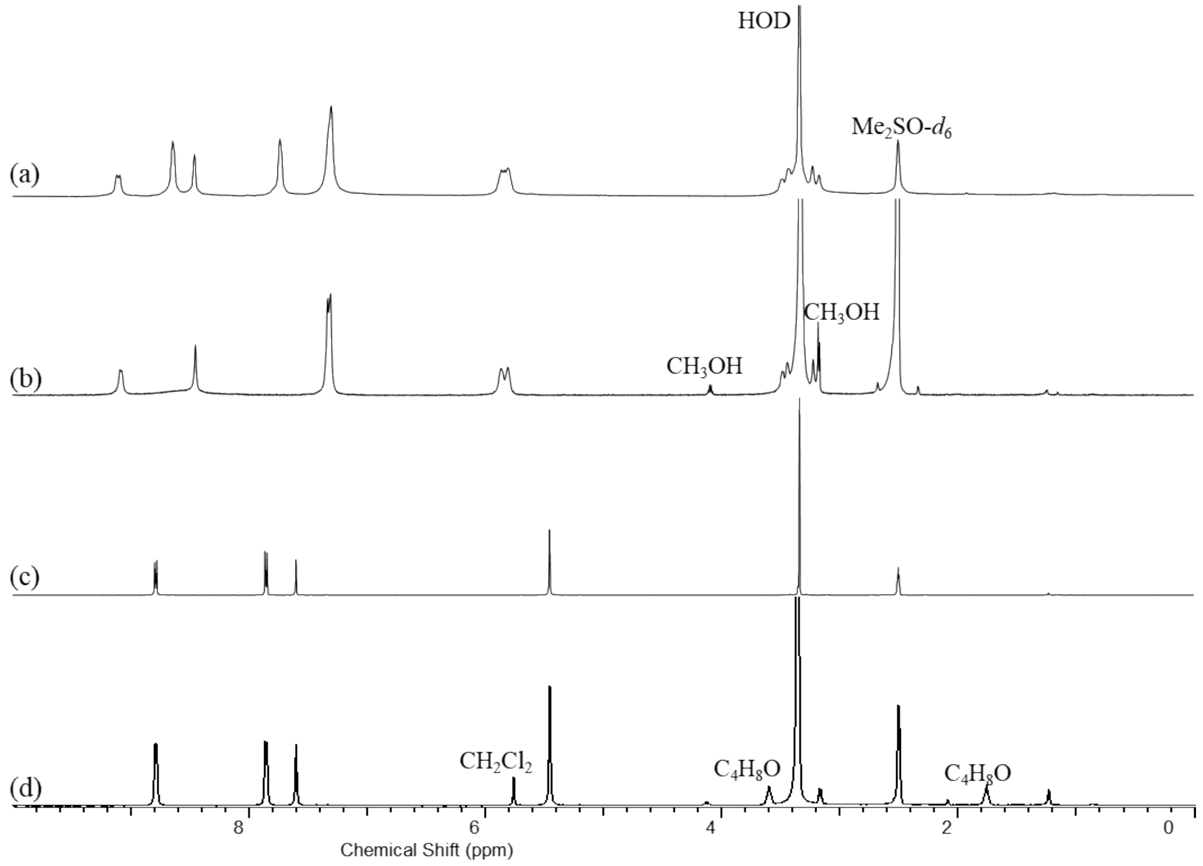

Fig. S4 ^{1}H NMR spectra for L^1 (a), $[(SiF_6)_2@Cu_3L^1{}_4](SiF_6)\cdot16CH_3OH$ (b), for L^2 (c), and $[(SiF_6)@Zn_2L^2{}_4](SiF_6)\cdot2C_4H_8O\cdot4CH_2Cl_2$ (d).

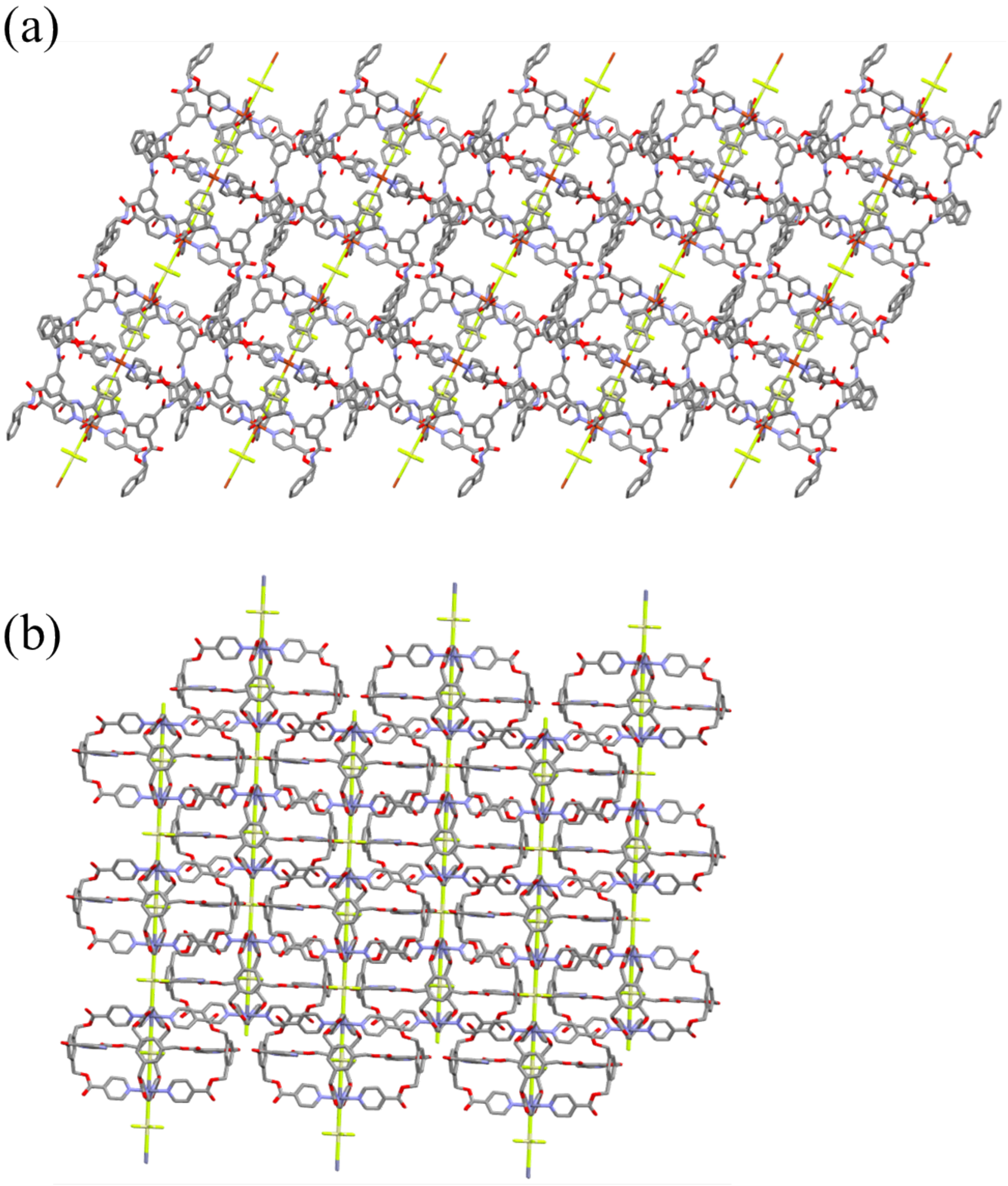

Fig. S5 The packing diagram represent the structure of $[(SiF_6)_2@Cu_3L^1{}_4](SiF_6)\cdot16CH_3OH$ (a), and $[(SiF_6)@Zn_2L^2{}_4](SiF_6)\cdot2C_4H_8O\cdot4CH_2Cl_2$ (b).

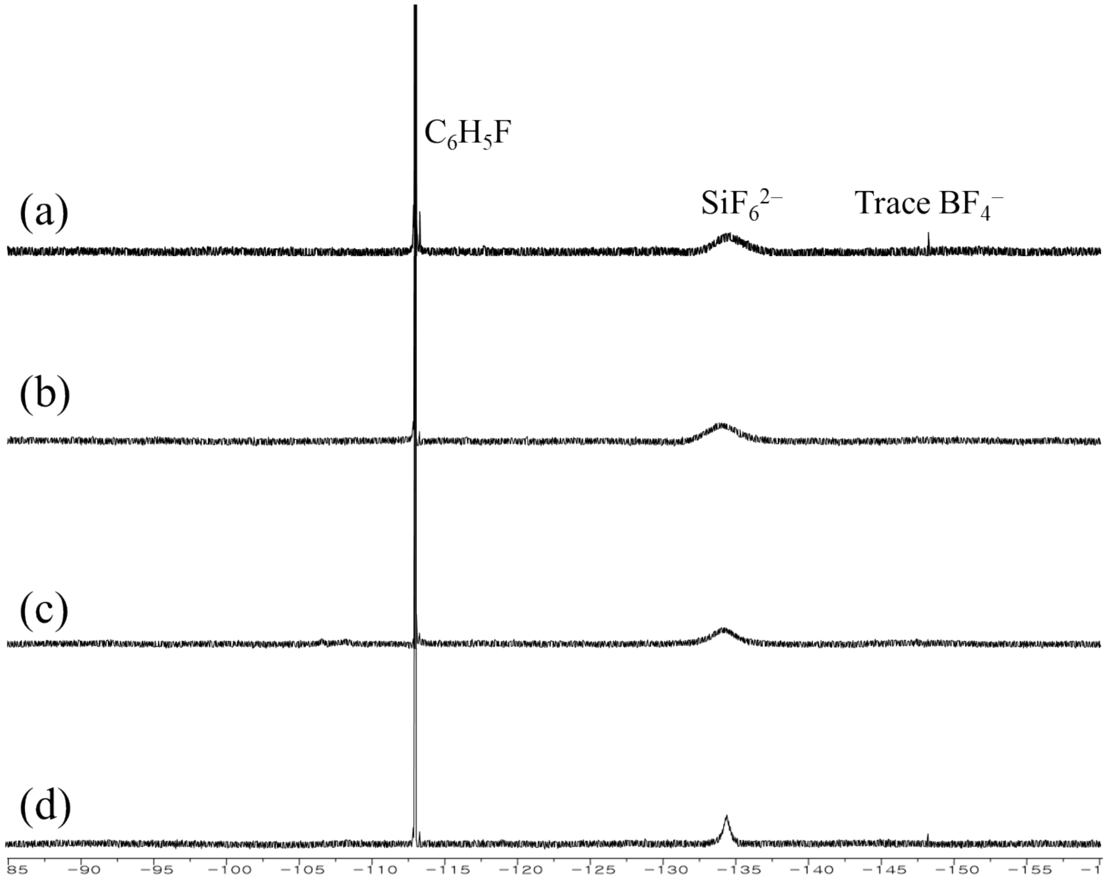

Fig. S6 ^{19}F NMR spectra for $[(SiF_6)_2@Cu_3L^1_4](SiF_6)\cdot16CH_3OH$ using *Method 1* (a), $[(SiF_6)_2@Cu_3L^1_4](SiF_6)\cdot16CH_3OH$ using *Method 2* (b), $[(SiF_6)_2@Cu_3L^1_4](SiF_6)\cdot16CH_3OH$ using *Method 3* (c), and $[(SiF_6)@Zn_2L^2_4](SiF_6)\cdot2C_4H_8O\cdot4CH_2Cl_2$ (d). The chemical shifts were measured relative to a C_6H_5F internal standard.
